# Fabrication and characterization of 3D printing scaffold technology by extract oils from plant and its applications in the cardiovascular blood

**DOI:** 10.1038/s41598-021-03951-z

**Published:** 2021-12-23

**Authors:** Soheila Naderi, Akbar Esmaeili

**Affiliations:** Department of Chemical Engineering, North Tehran Branch, Islamic Azad University, P.O. Box 1651153311, Tehran, Iran

**Keywords:** Drug discovery, Chemistry

## Abstract

Extract oils from plants used in 3D polysaccharides modified with natural protein polymer modified polymer scaffolds can help to reduce blood pressure. This study aimed to use extract oils from plant (EOP)as blood pressure-reducing, bind them to magnetic iron nanoparticles (Fe_3_O_4_@NPs), then bind them to polymeric 3D print scaffolds [chitosan, polylactic acid, and polyurethane (CS/PLA/PU), modified with natural protein and finally separate them. This method made it possible to investigate different variables for nanoparticles. In this project, synthesis polymer, modified gelatin (Mo-Ge), PEGylation, extract oils from plant loading and release process in nanocarrier with different concentrations were examined and cell proliferation was optimized. The results show that 75% of the extract oils from plant loaded on iron magnetic nanoparticles containing PEGylated polymer scaffolds was released. Cell proliferation was performed for the sample. In this process, modification of scaffolding with polysaccharides modified with natural protein and extract oils from plant increased the efficiency of nanoparticles among the studied *Allium sativum* and *Zingiber officinale*. The size of *A. sativum* and *Z. officinale* were 29.833 nm and 150.02 nm size, respectively. These behaved very similarly to each other and *A. sativum* had the biggest effect in lowering blood pressure. The application of extract oils from plant in 3D mode scaffolding has not been studied before and this is the first analysis to do so, using nanoparticles.

## Introduction

**Figure 1:**
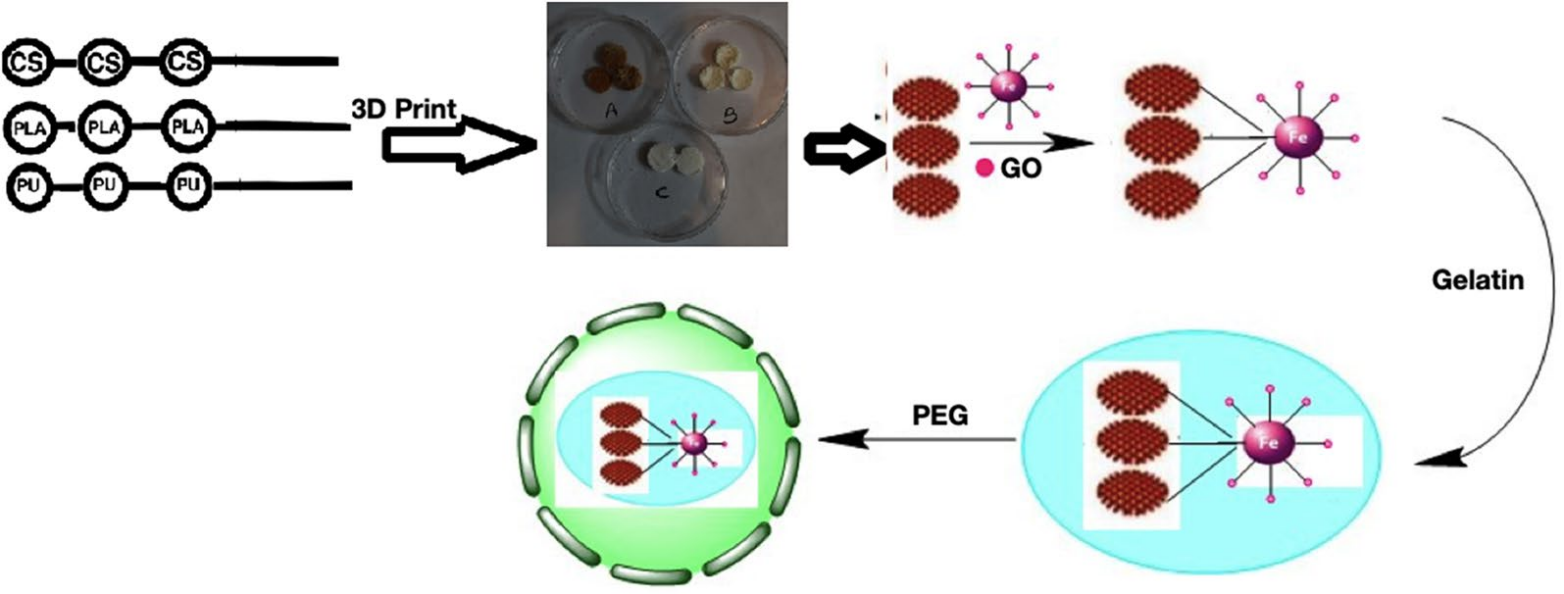
Process for modifying the surface of 3D print scaffolds [(Fe_3_O_4_@EOP/CS/PLA/PU-Mo-Ge) PEGylated] used in cardiovascular blood.

Today, there is a growing demand for woven vascular engineering, that is effective in the long-term for replacing or bypassing damaged arteries in various cardiovascular diseases. Ideal tissue engineering vessels should be biocompatible, blood compatible, and resistant to the spread of aneurysms, and be easily implantable in the body^1,2^. Research has shown that scaffolds are made from degenerated natural tissue or biodegradable biopolymers and synthetic polymers to make vascular bonds for tissue engineering. Polysaccharides are biological polymers that play a key role in improving health. PLA is considered to be one of the most widely used materials in 3D printing. Rapid decomposition in the environment (in just a few years) and use in medical products are among the features of this valuable substance^3^. The use of biocompatible and non-toxic substances chemically improves artificial scaffolds and improves their mechanical properties, which is one of the requirements for the production of ideal vascular grafts^4^. Gelatin is a solid, semi-transparent, and insoluble substance derived from bovine bone or collagen in pig skin, and because of its resemblance to collagen and its biological origin, it has been introduced as an attractive polymer for tissue engineering applications^5^. The reason for using CS for biocompatibility is biodegradability and non-toxicity. PLA in the body can be easily decomposition. For eliminate the disadvantages of this polymer, modified modes for making vascular scaffolds are considered. High elasticity and acceptable biocompatibility make PU an attractive material for vascular scaffolding^3^. Ganji et al.,^6^ used castor oil and polyethylene glycol to produce a plant-based scaffold that was able to produce blood vessels in the animal phase without regurgitation and infection under rabbit skin. Mozumder et al.,^7^ studied biomedical applications of polymeric nanobiocomposites. Pourfarhangi et al.,^8^ proposed a new way to build a polymer scaffold consisting of a decomposed cell network of the heart for use in cardiac tissue engineering. Khalili and Esmaeili^3^ were able to synthesize PU for use in tissue engineering. They explained hybrid polymer properties of nanofibers in anticoagulant drugs. O'Brien^9^ explained different materials application in scaffolds concerning the tissue engineering field. Tissue engineering with 3D printing can produce and repair damaged tissues engineering by combining cellular parts of the body with biocompatible materials. An et al.,^10^ explained that—compared to synthetic polymers 3D technical natural polymers can provide good biocompatibility for cells. The ability of natural polymers to print in 3D scaffolds is usually weak so for this reason, indirect 3D printing was created for porous 3D scaffolds. The reason of compositions of scaffold for the process, usage the abilities of each in this scaffolding. Two EOP of *A. sativum* and *Z. officinale* have been selected from among the eight EOP that are effective in regulating blood pressure^11^. *A. sativum* reduces the risk of heart disease by managing high cholesterol and blood pressure. *Z. officinale* can help lower blood cholesterol levels and prevent blood clots. By lowering cholesterol levels and blood clotting, the risk of clogged arteries is reduced and the risk of heart attacks and blood pressure. In fact, the main purpose of preparing blood vessels with suitable polymers in the form of 3D printing and placing drugs that regulate blood pressure on them. Among the EOP that are effective in regulating blood pressure, experiments were performed on eight EOP^12^. The results indicate that *A. sativum* and *Z. officinale* show the most similar behavior in regulating blood pressure, which confirms the similarity of the FT-IR and XRD spectra of these two drugs. Previous studies^13,14^ have shown that they are similar in terms of therapeutic effects they can have on the body. Because the investigation of analysis spectra was more pronounced in the samples containing *A. sativum* and *Z. officinale.* The study continued our research on it. The amount of drug loaded and the amount of drug released were measured in the same way as in the previous article and good results were obtained^15^. Figure 1 show preparation of 3D print scaffolds of [(Fe_3_O_4_@EOP/CS/PLA/PU-Mo-Ge) PEGylated].

## Materials and methods

### Materials

All laboratory materials used in this project, including chitosan [M_w_ = 1,250,000 (avg.), HS code: 3913900099, 75–85% degree of deacetylation], polylactic acid (medium molecular weight), polyurethane (medium molecular weight), polyethylene glycol (low molecular weight, 400), Tween 80 (26 kDa) were purchased from Sigma Chemical Co. Meanwhile, MnCl_2_.4H_2_O (98%), FeCl_3_.6H_2_O (98%), NaOH (99%), and glutaraldehyde (GA) were bought from Merck Co. Lastly, chloroform (99%) and formic acid (95%) solvents were sourced from Sigma-Aldrich, Germany.

### Synthesis of magnetic iron oxide nanoparticles

307.2 g of hexahydrate of iron chloride, 97.7 g of short-grained iron chloride were dissolved in 100 ml distilled water to make magnetic NPs. The subsequent product was placed in a bathroom sonicator for 30 min, then stirred for 2 h under a stream of nitrogen gas. Concentrated ammonia was used as the precipitating agent. Following the reaction, the sediments were separated by a magnetic separation method with a 1.3 T magnet (and washed with distilled water and ethanol). The sediment was then dried in an oven at 40 °C.

### Connection of *A*. *sativum *and *Z*. *officinale* to magnetic nanoparticles

In order to take advantage of the antihypertensive properties of EOP, various EOP were used, including olive oil, sesame, coconut, almond, *A. sativum* and *Z. officinale*, lavender and coriander. In the section used eight oil, but two oil suitable selected for the work. In this section, 0.5 g of eight each oil mixed with 0.5 g of Fe_3_O_4_ nanoparticle with formic acid for 24 h. After this the obtained products were extracted. Fe_3_O_4_@*A. sativum* and Fe_3_O_4_@*Z. officinale* formed in the section.

### Connection of Fe_3_O_4_@***A. sativum*** and Fe_3_O_4_@***Z. officinale*** to CS/PLA/PU solutions for scaffolding

For preparation CS/PLA/PU scaffolding was done using solutions with concentrations of 4–8%. The connection of CS/PLA/PU to Fe_3_O_4_@*A. sativum* and Fe_3_O_4_@*Z. officinale* scaffolding was obtained from a solution of 6% of oils, which was prepared from 2 g of polymer in 3.33 ml of formic acid. In the process Fe_3_O_4_@*A. sativum*/CS/PLA/PU and Fe_3_O_4_@*Z. officinale*/CS/PLA/PU were produced.

### Scaffolding method

6% solutions of each polymer (CS, PLA, and PU) were scaffolded with a 3D printer and kept at 80 °C temperature for 10 days. After the solvent dried, they were placed in the freezer for 5 days.

### Stabilization of scaffolding

In order to establish a connection between the molecules, CS/PLA/PU scaffolds were placed in a 2% volumetric GA solution for 4 h. They were then removed from the solution and rinsed with deionized water and finally placed in a freezer for 24 h.

### Preparation of 3D print scaffold Fe_3_O_4_@***A. sativum*** /CS/PLA/PU and Fe_3_O_4_@***Z. officinale***/CS/PLA/PU

3D printer scaffolds were prepared from each of 6% solutions of CS, PLA, and PU. The scaffolds were kept at 8 °C for 10 days. Until their solvent dries. They were placed in the freezer for 5 days.

### Modification of 3D print scaffold containing Fe_3_O_4_@***A. sativum***/CS/PLA/PU and Fe_3_O_4_@***Z. officinale***/CS/PLA/PU by gelatin

In order to improve the adhesion of the cell to the scaffold’s surface and to increase its surface properties for use in tissue engineering, 3D print scaffold of CS/PLA/PU containing magnetic iron NPs and drug was reacted with gelatin. In this way, 1 g from the scaffold was mixed with 0.5 g gelatin in 100 ml water for 24 h and then the resulting sediments were collected. In the process 3D print scaffold modified with gelatin Fe_3_O_4_@*A. sativum*/CS/PLA/PU-Mo-Ge and Fe_3_O_4_@*Z. officinale*/CS/PLA/PU-Mo-Ge were produced.

### PEGylation of 3D print scaffold containing Fe_3_O_4_@***A. sativum***/CS/PLA/PU-Mo-Ge and Fe_3_O_4_@***Z. officinale***/CS/PLA/PU-Mo-Ge

To increase the stability and durability of scaffolds in the body, scaffolds were PEGylated. In such a way 0.5 g PEG (400) is dissolved in 100 ml of 98% ethanol (ambient temperature, 300 rpm). This solution is fixed on pH = 7.4 by 1 M sodium hydroxide. The scaffolds containing magnetic iron NPs and drug were mixed with this solution and placed at room temperature for 18 h. Then, the PEGylated scaffolds were separated by 1.3 T magnet and distilled with water and ethanol washed twice. In the process Fe_3_O_4_@*A. sativum*/CS/PLA/PU modified gelatin PEGylated [(Fe_3_O_4_@EOP/CS/PLA/PU-Mo-Ge) PEGylated].

### Scanning electron microscopy (SEM) analysis

The morphology and measurement analysis of the nanoparticle was investigated in condition of SEM with model of KYKY EM3200 Digital SEM, China. The observation of nanoparticle under SEM system, nanoparticles were prepared in the form of sediment by spraying on a gold foil with aluminum foil system (KYKY SBC-12 Ion Sputtering Coater).

### XRD analysis

XRD (Siemens D 5000 X-ray Powder Diffraction System, Germany), is a rapid analytical technique primarily used for phase identification of a crystalline material and can provide information on unit cell dimensions. The XRD analyzed material is finely ground, homogenized, and average bulk composition was determined. XRD using copper beams at 25 °C, at a rate of degrees per second, on a scale of 2θ and in one step of 1 s. The average crystalline size was obtained from X-ray diffraction data using Scherrer’s formula (Eq. 1):

1 $$D=\frac{K\lambda }{\beta {\text{cos}}\theta }$$

where k = 0.94, λ = 0.154056 nm, and β is the full width at half maximum in radians^16,17^.

### FT-IR analysis

FT-IR microscope (Thermo Scientific Inc., USA) with liquid nitrogen cooled MCT-A detector has been used, conical shape germanium tip crystal (350-micron spherical finish, single reflection, throughput > 50%, 27° average angle). Each spectrum has been achieved in the range of 4000–650 cm^−1^ at a spectral resolution of 8 cm^−1^ and with 128 scans on the average using Omnic software (Thermo Sci- entific Inc., USA).

### Release of medication

#### Determining the actual amount of EOP loading

Ultraviolet–visible (UV–Vis) spectroscopy at 275 nm served to determine the actual amount of EOP (*A. sativum* and *Z. officinale*) loaded on magnetic iron NPs containing PEGylated polymer scaffolds (CS/PLA/PU). *A. sativum* and *Z. officinale* oils show absorption at 275 nm. 50 mg of the sample (Resolved by diffusion method in minimums of formic acid) was dissolved in 100 ml of the phosphate buffer pH = 7.4 and stirred for 24 h at room temperature with a magnetic stirrer. Then the product was separated. The actual amount of EOP (*A. sativum* and *Z. officinale*) loaded was calculated by the following equation (Eq. 2):

2 $$LC\%=\left(\frac{weight \; of \; loaded \; EOP}{weight \; of \; sample}\right)\times 100$$

#### Determination of EOP release profile

40 mg of the EOP (*A. sativum* and *Z. officinale*) was dissolved in 100 ml of phosphate buffer solution with pH = 7.4 and stirred at room temperature with a magnetic stirrer. With a certain time, interval of up to 48 h, 5 ml from the top solution was removed and the sediments in it were separated. Then its absorption was read by a UV–Vis device at 275 nm and the amount of EOP was obtained from the calibration curve. The EOP cumulative percentage was calculated from the following equation (Eq. 3):

3 $$cumulative \; release \; rate=\sum_{t=0}^{t}\frac{Mt}{M0}\times 100$$

where it is the EOP (*A. sativum* and *Z. officinale*) release time, Mt is the cumulative amount of EOP in time unit and M0 is the initial amount of EOP (*A. sativum* and *Z. officinale*) in the sample.

### Cell viability

Dimethyl thiazole 2, 5-diphenyltetrazolium bromide (MTT) assay is used to evaluate the bioavailability of cells. This method is the reduction of the yellow salt of dimethyl thiazole diphenyltetrazolium bromide into insoluble and purple crystals of formazan dye. It is carried out by living cell mitochondrial dehydrogenase enzymes. The intensity of the purple dye produced is directly related to the amount of cells that are metabolically active. 5 mg of thiazolyl blue tetrazolium bromide powder was dissolved in 1 ml of PBS solution. The resulting solution was passed through a sterile Falcon filter of 0.22 microns and wrapped in foil. After counting the cells, an appropriate number of cells were cultured in the desired cells from a 96-cell plate with a final volume of 200 μl of serum medium. Place the plate in the incubator for 24 h to allow the cells to adhere to the surface of the plate. Pour the right amount of NPs into each well and pipette well. Each concentration is tested in triplicate. After the desired time, the wells were completely emptied and 180 μl of culture medium and 20 μl of MTT solution were added to each well. The plate was wrapped in foil and incubated at 37 °C for 4 h. The medium was removed from the wells and 200 μL of DMSO was added to each well. After 10 min, the light absorption of the samples was read at 570 nm. In this study, MTT assay was performed for cancer cells as well as normal cells within 24 h after treatment with test groups. The results show that there is no significant difference with the control group without drugs and their combination does not show the effect of cytotoxicity.

## Results and discussion

### FT-IR analysis

**Figure 2:**
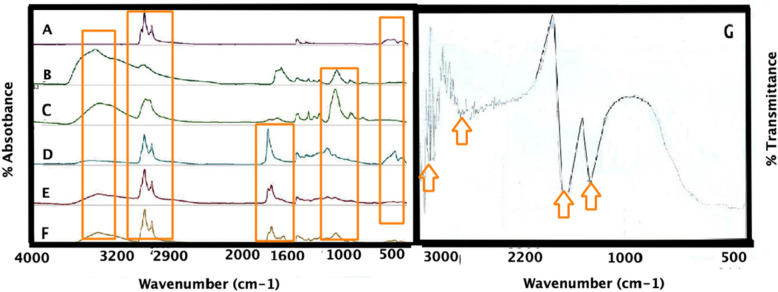
Comparison of the FT-IR spectra of (**A**) Fe_3_O_4_@*A. sativum* (**B**) Fe_3_O_4_@*A. sativum*/CS/PLA/PU; (**C**) (Fe_3_O_4_@*A. sativum*/CS/PLA/PU-Mo-Ge) PEGylated; (**D**) Fe_3_O_4_@*Z. officinale*; (**E**) Fe_3_O_4_@*Z. officinale*/CS/PLA/PU; (**F**) (Fe_3_O_4_@*Z. officinale*/CS/PLA/PU-Mo-Ge) PEGylated (all samples was liquids); (**G**) FT-IR of polymer scaffolds without addition of nanoparticles (the sample was powder).

FT-IR analysis helped to characterize chemical interaction of iron magnetic NPs connected *A. sativum* and *Z. officinale*, after connecting with polymeric scaffolds (CS/PLA/PU), and after the PEGylation, in the 400–4000 cm^−1^ range [see Fig. 2 (all samples was liquids)]. FT-IR spectrum of iron magnetic NPs connected to *A. sativum* (Fig. 2A), after connecting with polymeric scaffolds (CS/PLA/PU) (Fig. 2B), and after the PEGylation (Fe_3_O_4_@*A. sativum*/CS/PLA/PU-Mo-Ge) (Fig. 2C), identified the chemical absorption (Fig. 2). FT-IR spectrum of iron magnetic NPs connected to *Z. officinale* (Fig. 2D), after connecting with polymeric scaffolds (CS/PLA/PU) (Fig. 2E), and after the PEGylation (Fe_3_O_4_@*Z. officinale*/CS/PLA/PU-Mo-Ge) (Fig. 2F), identified the chemical absorption (Fig. 2). The results show that the *A. sativum* and *Z. officinale* exhibited the closest behavior to other oils (including olive oil, sesame, coconut, almond, *A. sativum* and *Z. officinale*, lavender and coriander). In Fig. 2A,D, the absorption of Fe–O of iron magnetic NPs is seen at 500 cm^−1^. The specific peaks at 476 and 578 cm^−1^ may be due to O-Fe stretching vibration and 1645 cm^−1^ is related to H_2_O deformation, respectively^18^. In Fig. 2B,E, the strong absorption of PLA and CS (C=O, C–O) are evident at 1560 cm^−1^ while the absorption of PU (C–H, C–O) is seen at 1340 cm^−1^^3^. Figure 2B in the FT-IR spectrum is related to the addition of CS/PLA/PU to Fe_3_O_4_@*A. sativum* and Fe_3_O_4_@*Z. officinale*. There are about 1100 cm^−1^ and C–O–C, 1600 cm^−1^ N–H bonds, 1700 cm^−1^ C=O, and about 3400 cm^−1^ O–H bonds, all of which belong to polymers. Furthermore, a broad absorption related to *A. sativum* can be seen at 3000 cm^−1^. The absorption of the N–H and O–H bonds exist at 3000 cm^−1^^19^. To confirm coating polymers on Fe_2_O_4_ NPs containing oil, broad absorption peaks at 3394 and 3417 cm^−1^ belong to the OH group of polymers. Absorption at 1617 cm^−1^ is due to the stretching vibrations C=O of polymers. In Fig. 2C, a strong absorbance at 1100 cm^−1^ is observed and it is related to the C–O–C bond of ethylene glycol. 3411 and 3382 cm^−1^ OH concerning the PEGylated polymers^20^. In Fig. 2G, FT-IR of polymer scaffolds without addition of nanoparticles (the sample was powder). In Fig. 2G the strong absorbance of PLA and Cs (C=O, C–O) are seen at 1560 cm^−1^ while the absorbance of PU (C–H, C–O) is seen at 1340 cm^−1^. Peak of the N–H and O–H bonds are in 3000 cm^−1^. A strong signal in 1100 cm^−1^ is seen and it is related to the C–O–C bond of ethylene glycol.

### XRD analysis

**Figure 3:**
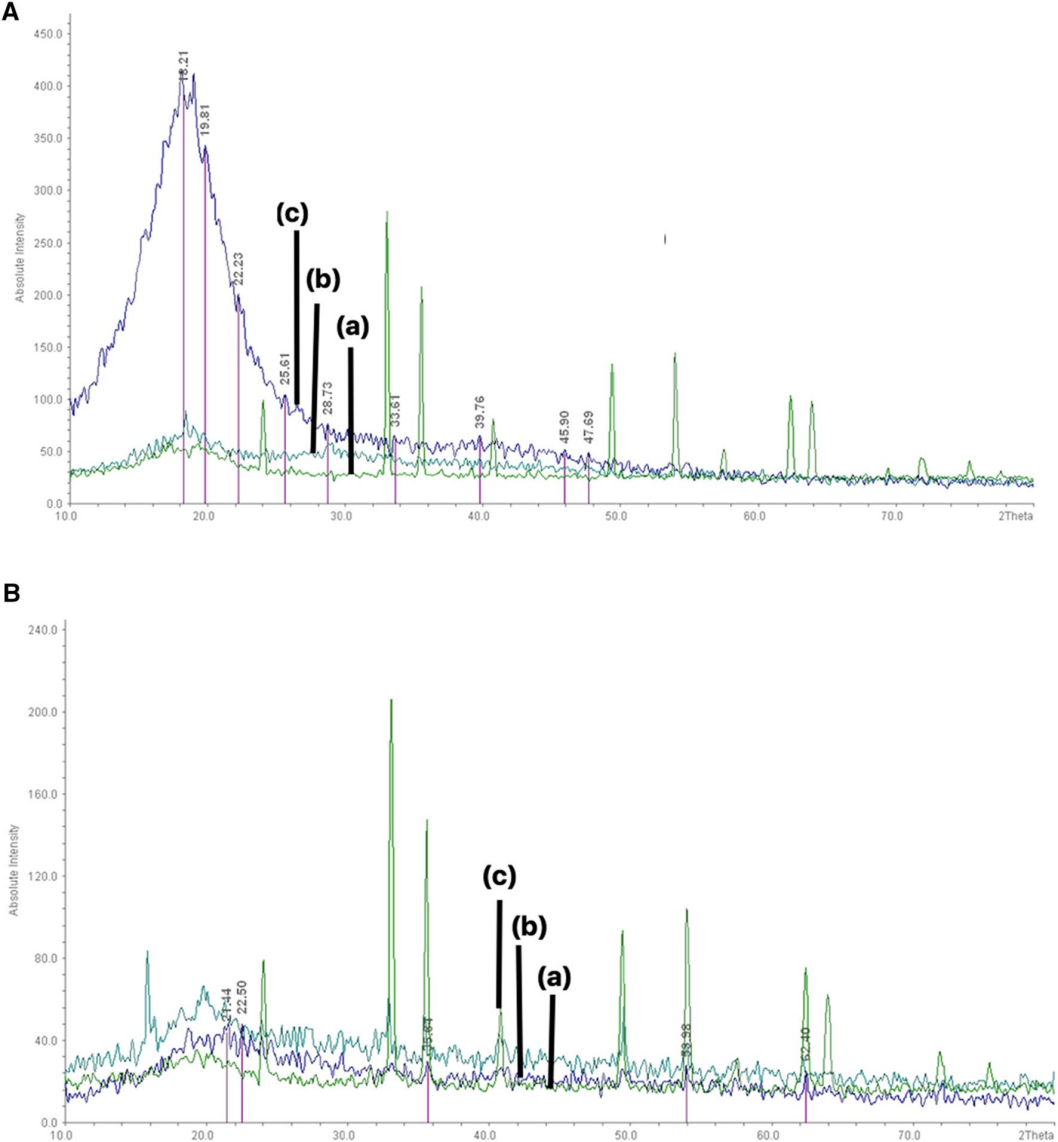
XRD pattern of (**A**) (a) Fe_3_O_4_@*A. sativum;* (b) Fe_3_O_4_@*A. sativum*/CS/PLA/PU; (c) (Fe_3_O_4_@*A. sativum* /CS/PLA/PU-Mo-Ge) PEGylated; (**B**) (a) Fe_3_O_4_@*Z. officinale;* (b) Fe_3_O_4_@*Z. officinale*/CS/PLA/PU; (c) (Fe_3_O_4_@*Z. officinale*/CS/PLA/PU-Mo-Ge) PEGylated.

For evaluate the appropriate particle size and morphology, XRD was used to confirm the results of SEM images. Figure 3A,B illustrate the crystalline structure of prepared [(Fe_3_O_4_@*A. sativum*/CS/PLA/PU-Mo-Ge) PEGylated] and [(Fe_3_O_4_@*Z. officinale*/CS/PLA/PU-Mo-Ge) PEGylated], which were confirmed by XRD analysis with 29.833 nm and 150.02 nm size, respectively. The diffraction peaks are related to the particles’ crystal structure in the angle range of (1 < 2θ < 80). Comparison of the XRD patterns of *A. sativum* and *Z. officinale* in the Fig. 3A,B indicates the most obvious changes in the XRD pattern of *A. sativum* (Fig. 3A). XRD pattern of Fe_3_O_4_@*A. sativum* [Fig. 3A(a)], after Fe_3_O_4_@*A. sativum*/CS/PLA/PU [Fig. 3A(b)], and [(Fe_3_O_4_@*A. sativum*/CS/PLA/PU-Mo-Ge) PEGylated] [Fig. 3A(c)], is show in Fig. 3A. The XRD patterns reveal absorption at 14 to 30 and these are related to polymers so it agrees with what previous studies have documented^21^. The results show that the *A. sativum* and *Z. officinale* indicate the closest behavior to other oils. Of these, *A. sativum* shows the most obvious changes (Fig. 3A,B). In Fig. 3A, which refers to the XRD analysis of the sample containing *A. sativum*, it clearly shows the changes in increasing both the NPs and CS/PLA/PU, and PEGylating the sample (Fig. 3A). The XRD patterns exhibited peaks corresponding to Fe_3_O_4_, marked with their indices (250), (355), (370), (454), (522) and (575), which are similar to those reported before for Fe_3_O_4_ NPs^22^. The XRD of patterns showed absorption at 14 to 30, related to CS/PLA/PU and this echoes previous studies^21^. The XRD patterns exhibited peaks corresponding to Fe_3_O_4_@*Z. officinale*/CS/PLA/PU, marked with their indices (220), (311), (400), (422), (511), and (440), which are similar to those reported previously for Cs/Fe_3_O_4_/NPs, and marked with 2θ = 16–29 and 2θ = 19, 20. These are similar to those reported before for PLA and PU, respectively. Figure 3B(a) show XRD of patterns showed absorption at 19 and 23, related to polyethylene glycol, after PEGylation, which is similar to previous studies^23^. XRD pattern of Fe_3_O_4_@*Z. officinale* [Fig. 3B(a)], after Fe_3_O_4_@*Z. officinale*/CS/PLA/PU [Fig. 3B(b], and [(Fe_3_O_4_@*Z. officinale*/CS/PLA/PU-Mo-Ge) PEGylated] [Fig. 3B(c)], is shown in Fig. 3B.

Figure 3B(a) show XRD patterns exhibited peaks corresponding to Fe_3_O_4_, marked with their indices (250), (355), (370), (454), (522), and (575), which are similar to those reported before for Fe_3_O_4_ NPs^24,25^. The XRD of patterns showed absorption at 14 to 30, related to polymers as indicated in other studies^26^. Figure 3B(b) show XRD patterns exhibited peaks corresponding to Fe_3_O_4_@*Z. officinale*/CS/PLA/PU, marked with their indices (220), (311), (400), (422), (511), and (440), which are similar to those reported before for CS/Fe_3_O_4_ NPs, and marked with 2θ = 16–29 and 2θ = 19, 20 which are similar to those reported before for PLA and PU, respectively^27^. Figure 3B(c) show XRD of patterns revealed absorption at 19 and 23, related to polyethylene glycol, after PEGylation, which is similar to previous studies^23^. The results show that *A. sativum* and *Z. officinale* behave very similarly to other oils and among these, *A. sativum* shows the most obvious changes [Fig. 3B(b,c)]. In Fig. 3A, which illustrates the XRD analysis of the sample containing *A. sativum*, it clearly depicts the changes involved in: firstly, increasing the NPs and the CS/PLA/PU; and secondly, PEGylating the sample.

### SEM analysis

**Figure 4:**
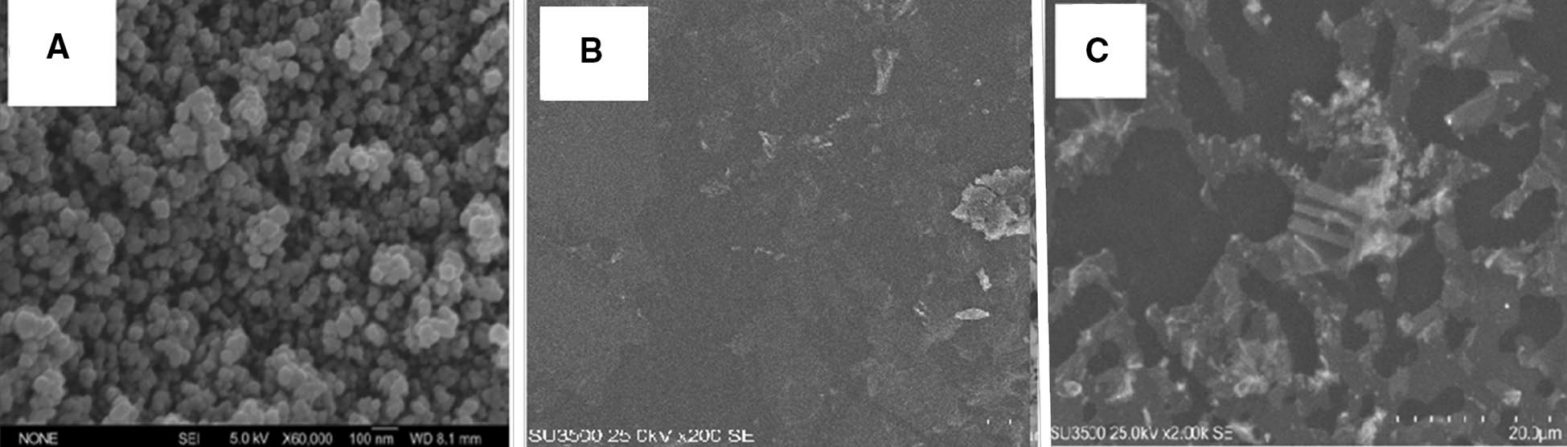
SEM pattern of (**A**) Fe_3_O_4_@*A. sativum*; (**B**) Fe_3_O_4_@*A. sativum*/CS/PLA/PU; (**C**) (Fe_3_O_4_@*A. sativum*/CS/PLA/PU-Mo-Ge) PEGylated.

Morphological analysis was undertaken with the help of electron microscopic images. Figure 4A–C shows the SEM images of the samples. Figure 4A relates to Fe_3_O_4_@*A. sativum* and Fig. 4B illustrates the combination of Fe_3_O_4_@*A. sativum*/CS/PLA/PU. Figure 4B shows the SEM image of Fe_3_O_4_@*A. sativum*/CS/PLA/PU, and it can be seen clearly that the particles are uniformly aggregated, spherical shaped with a size of 50–350 μm^2,29,30^. Figure 4B shows the PEGylation of the 3D print scaffold (CS/PLA/PU) attached to the magnetic iron NPs containing the gelatin-coated *A. sativum*. Figure 4C corresponds to the combination [(Fe_3_O_4_@*A. sativum*/CS/PLA/PU-Mo-Ge) PEGylated]. In the SEM image of iron magnetic NPs connected to Fe_3_O_4_@*A. sativum*, it can be seen clearly that the particles are uniformly aggregated, spherical shaped with a size of 6–30 nm^28^. In the section entered SEM of *A. sativum*. Because the SEMs of the *A. sativum* and *Z. officinale* oils were similar. In this study we attempted to examine the image with an electronic microscope. Since the sample was very thick and oily and did not dry completely even under vacuum, we had to dissolve it in a little methanol and then take a picture of it. Measurement of the sample when using SEM revealed that the sample was 100–135 nm in size.

### ZPS analysis

The ZPS represents the electric charge on the membrane surface. The ions in the environment as well as the pH of the environment affect ZPS amount. In this experiment, ZPS, deviation and guidance were obtained as − 1.29, 0, and 13.3, respectively. These values indicate that the polymers used in the construction of the scaffold have good conductivity in solution and are also blood compatible. Therefore, they can be effective in in vivo testing. The particle has a surface charge inside the fluid, and an increase in the concentration of ions with the opposite charge to the surface of the particle is always seen around the surface of the particle inside the fluid. Thus, an additional layer of these ions surrounds the surface of the particle and forms an additional layer around the particle. When a particle moves in a fluid, the surrounding layer also moves with the particle and moves with the particle, and it can be assumed that a hypothetical distance between the particle and the fluid environment is the hypothetical distance of the extra layer that surrounds the particle. This distance is called the hydrodynamic distance and the potential at this distance is known as the zeta potential. In fact, the zeta potential is a parameter for the potential stability of the colloidal system. If all the particles in the suspension are negatively or positively charged, the particles tend to repel each other and show no tendency to coalesce.

The tendency of particles to repel each other is directly related to the zeta potential. In general, the limit of the suspension’s stability and instability can be determined in terms of zeta potential. Esmaeili and Khodaei^31^ reported the negative ZPS of PU. They showed PU can be used as a conductive solution in the double-needled electrospinning method. The negative charge of the PU surface led to a better conduction of ions in the electrospinning device. It also caused better blood compatibility with PU than other polymers. They showed that the negative and positive numbers obtained can be effective in clinical tests^3^. So, we decided to do this test on our sample that contained the three polymers (CS/PLA/PU). The results show that the zeta potential is -1.29 mV, the zeta deviation is 0 mV and the ion conductivity is 13.3 ms/cm.

### In vitro release of EOP (*A. sativum *and *Z. officinale*) loaded

**Figure 5:**
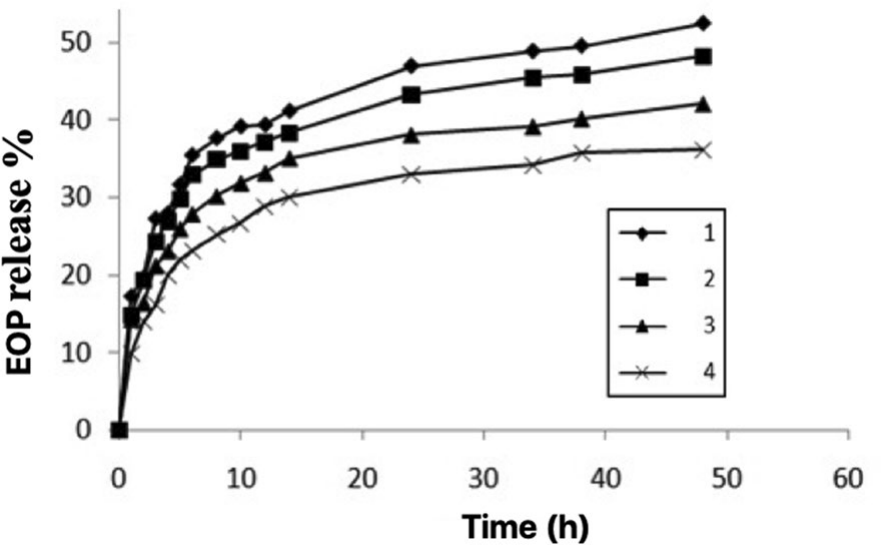
The different ratio scaffolds/EOP in 1, 2, 3, and 4 with loading EOP is 52.1, 36.2, 28.3, and 24.4%.

Figure 5 show in vitro release profiles of EOP (*A. sativum* and *Z. officinale*) from nanoparticle loaded were investigated in 100 ml of phosphate buffer solution with pH = 7.4 in 48 h. The amount release by UV–Vis spectrophotometry at λ = 275 nm in different ratio scaffolds/EOP (1, 2, 3, and 4) with percent of loading EOP (52.1, 36.2, 28.3, and 24.4%). The higher the ratio of CS/PLA/PU to EOP, the lower the amount of EOP loaded. Therefore, the best formulation is related to the case in which the ratio of CS/PLA/PU to EOP is 1: 1 and has the highest drug load. The release of oils from nanoparticles is done by various mechanisms such as decomposition, desorption and diffusion^32^.

The mechanism of oil release from the matrix is done by releasing the oil out of the nanoparticles into the environment^33^.The pattern of EOP from nanoparticle release shows a very slow release. For the first initial until final process shows that 75% of the EOP loaded on iron magnetic NPs containing CS/PLA/PU scaffolds was released. The release of EOP at this stage is due to the binding of EOP molecules attached to the surface of the nanoparticle and near the surface of the nanoparticles^34^. It can be attributed to the diffusion of EOP and dispersion in nanoparticles due to penetration into the buffer solution, which causes the bonded CS/PLA/PU with EOP^35,36^. The release of more EOP in the neutral medium may be attributed to the dissolution of the CS/PLA/PU due to the ionic repulsion of the amino groups present in the CS, PLA, and PU and the binding to other groups^37,38^. In previous research, increase in release to pH conditions related. The release of curcumin from nanoparticles was related to an acidic environment with pH = 5^38^.

### Cell viability

In order to evaluate the survival of human fibroblast cells under the influence of [(Fe_3_O_4_@*A. sativum* and *Z. officinale*/CS/PLA/PU-Mo-Ge) PEGylated], MTT test was undertaken. Therefore, human fibroblast cells were cultured in appropriate numbers for this test in 24 h after 3D print scaffolds formation. The cells were then treated with a range of drug concentrations in triplicate.

After reading the final absorption by ELISA reader, it was considered to be 100% and other samples were weighed against it. Esmaeili and Hormozi^39^ synthesized magnetic NPs of albumin with organic compounds for absorbing and releasing doxorubicin hydrochloride and then investigated the toxicity of NPs by MTT test. Esmaeili and Khalili^3^ prepared a scaffold made of CS//PVA/PU with double-needle electrospinning. It contained anticoagulant drugs and we then examined its toxicity using an MTT test. In this study, MTT assay was performed for cancer cells as well as normal cells within 24 h after treatment with the test groups. Table 1 shows the results of light absorption of the studied groups for MTT solution in a period of 24 h for 3D print scaffolds of [(Fe_3_O_4_@*A. sativum*/CS/PLA/PU-Mo-Ge) PEGylated] and [(Fe_3_O_4_@*Z. officinale*/CS/PLA/PU-Mo-Ge) PEGylated]. Each 3D print scaffolds of [(Fe_3_O_4_@*A. sativum*/CS/PLA/PU-Mo-Ge) PEGylated] and [Fe_3_O_4_@*Z. officinale*/CS/PLA/PU-Mo-Ge) PEGylated] concentration was repeated three times.

**Table 1:** Light absorption of the 4 groups for MTT solution in a period of 24 h.

Concentration (MG ML^−1^)	1	2	3	Control group (NPS without EOP)
1.2048	0.881	0.784	0.891	0.861
1.1024	0.799	0.751	0.850	0.747
1.5120	0.645	0.708	0.612	0.824
1.2560	0.202	0.215	0.232	0.791
1.1280	0.006	0.005	0.025	0.822

Each drug concentration was repeated three times.

**Figure 6:**
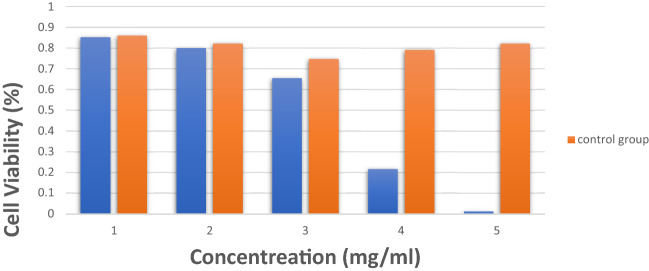
Cell proliferation test (MTT) for [(Fe_3_O_4_@EOP/CS/PLA/PU-Mo-Ge) PEGylated], and Control group (NPs without EOP).

Figure 6 show cell proliferation test for [(Fe_3_O_4_@EOP/CS/PLA/PU-Mo-Ge) PEGylated]. The results show that *A. sativum* and *Z. officinale* used in five concentrations is not significantly different from the control group without the 3D print scaffolds of [(Fe_3_O_4_@*A. sativum*/CS/PLA/PU-Mo-Ge) PEGylated] and [(Fe_3_O_4_@*Z. officinale*/CS/PLA/PU-Mo-Ge) PEGylated] and the combination does not show a cytotoxic effect.

## Conclusion

In this study, the CS/PLA/PU were formed into three-3D print scaffolds. Fe_3_O_4_@EOP were attached to these scaffolds and finally their assembly was modified with Fe_3_O_4_@EOP/CS/PLA/PU-Mo-Ge. The finally was PEGylated [(Fe_3_O_4_@EOP/CS/PLA/PU-Mo-Ge) PEGylated]. The best scaffolding was obtained from the concentration of 6% of each polymer in formic acid. In this method, eight vegetable oils were used, all of which are effective in regulating blood pressure. The results showed that *A. sativum* and *Z. officinale* oils show similar behaviors. So, we focused most of our work on *A. sativum*, which showed the best response. EOP used as blood pressure regulators and engineering of cardiovascular tissue. *A. sativum* and *Z. officinale* oils show absorption at 275 nm. Therefore, using the UV–Vis spectra, we found that the EOP load on the magnetic iron nanoparticles attached to the 3D scaffold is 75%. Meanwhile, the pH of the working environment was kept at 7.4. Examination of the FT-IR and XRD spectra showed that the 3D print scaffolds of EOP had not chemical interaction. With MTT tests, the absence of cytotoxicity in this system was investigated. The experiment was repeated 3 times in 5 different concentrations with a ratio of about 0.9 for the sample and the control group. It is not clear difference between the sample and the control group, the non-toxicity of the sample was proven. This work is medically biocompatible with blood in the presence of [(Fe_3_O_4_@EOP/CS/PLA/PU-Mo-Ge) PEGylated] due to the structural similarity of CS/PLA/PUwith the layers of blood vessels. It was considered an effective option for cardiovascular systems.
